# Early Rectal Cancer: Advances in Diagnosis and Management Strategies

**DOI:** 10.3390/cancers17040588

**Published:** 2025-02-09

**Authors:** Huda Mohammed, Hadeel Mohamed, Nusyba Mohamed, Rajat Sharma, Jayesh Sagar

**Affiliations:** 1 Surgery Department, Colorectal Surgery, Luton and Dunstable Hospital, Luton LU4 0DZ, UK; huda.mohammed@bedsft.nhs.uk (H.M.); nusyba.mohamed@ldh.nhs.uk (N.M.); rajat.sharma@bedsft.nhs.uk (R.S.); 2 Faculty of Medicine, University of Khartoum, Khartoum 11115, Sudan; tota16016@gmail.com

**Keywords:** early rectal cancer, local resection (LR), total mesorectal excision (TME), endoscopic mucosal resection (EMR), endoscopic submucosal dissection (ESD), transanal endoscopic microsurgery (TEM), transanal minimally invasive surgery (TAMIS), transanal endoscopic operations (TEO)

## Abstract

Colorectal cancer (CRC) is the second most prevalent cause of cancer-related death and the third most common cancer globally. Early-stage rectal cancer is defined by lesions confined to the bowel wall, without extension beyond the submucosa in T1 or the muscularis propria in T2, with no indication of lymph node involvement or distant metastasis. The gold standard for managing rectal cancer is total mesorectal excision (TME); however, it is linked to considerable morbidities and impaired quality of life. There is a growing interest in local resection and non-operative treatment of early RC for organ preservation. Local resection options include three types of transanal endoscopic surgery (TES): transanal endoscopic microsurgery (TEM), transanal endoscopic operations (TEO), and transanal minimally invasive surgery (TAMIS), while endoscopic resection includes endoscopic mucosal resection (EMR), underwater endoscopic mucosal resection (UEMR), and endoscopic submucosal dissection (ESD). Although the oncological outcome of local resection of early rectal cancer is debated in the current literature, some studies have shown comparable outcomes with radical surgery in selected patients. The use of adjuvant and neoadjuvant chemoradiotherapy in early rectal cancer management is also controversial in the literature, but a number of studies have reported promising outcomes. This review focuses on the available literature regarding diagnosis, staging, and management strategies of early rectal cancer and provides possible recommendations.

## Simple Summary

Colorectal cancer is a serious health issue that affects millions of people worldwide and requires careful diagnosis and treatment. The good news is that when diagnosed early, colorectal cancer can be treated effectively. However, the treatment options can vary greatly depending on several factors, including the patient’s age, overall health, and the stage and type of cancer. Therefore, we conducted this review to gather information from current medical research and provide a clear understanding of the different ways to diagnose and treat early-stage rectal cancer so that patients can receive the most effective care possible and improve their recovery. We also wanted to highlight some of the controversies surrounding the treatment of this disease. Ultimately, our aim is to empower patients and healthcare providers with accurate and up-to-date information about the diagnosis and treatment of early-stage rectal cancer. By doing so, we hope to contribute to better health outcomes and improved quality of life for those affected by this disease.

## 1. Introduction

Colorectal cancer (CRC) is the second most prevalent cause of cancer-related mortality and the third most common cancer globally [1]. Of the approximately 1.8 million CRC cases that happen annually, 704,000 are diagnosed in the rectum [2]. After the implementation of CRC screening programs, the rate of early-stage CRC detection has increased dramatically from 5% to 17% [3,4]. A study utilizing data from the United States Surveillance, Epidemiology, and End Results program found that while CRC incidence is declining by 3.1% annually in individuals over 50 years old, the incidence of early-onset CRC in patients aged below 50 years is rising by 1.4% per year for unclear reasons [5,6]. Inequities in rectal cancer (RC) incidence and outcomes are evident within the early-onset population, with Black and Hispanic individuals experiencing higher rates of the disease compared to other groups [7,8]. Furthermore, younger patients are more likely to present with aggressive forms of RC and might require emergency surgical interventions. Despite these challenges, patients with early-onset RC often achieve comparable surgical outcomes to late-onset patients [5,7].

Early-stage rectal cancer refers to any malignant lesions that remain confined to the mucosa, submucosa, and muscularis propria [9]. Colorectal adenomas can transform into carcinoma depending on the histopathological type of adenoma. The incidence of transformation into invasive carcinoma is reported in up to 30% of villous adenomas but in only about 4% of tubular adenomas [10].

The standard treatment model is radical surgery, but it often leads to high rates of permanent stomas and a wide range of morbidities [11,12]. The management of rectal cancer has considerably evolved by incorporating neoadjuvant chemoradiotherapies and focusing on organ-preservation approaches, including surgical and non-surgical options for the curative treatment of rectal cancer [11,13].

Although the introduction of local excision has provided an alternative management option for early rectal cancer, local and nodal recurrence after such excision has always been a concern [14,15].

This review focuses on discussing variations in staging, diagnostic difficulties, management strategies, and outcomes and provides possible recommendations.

## 2. Staging of Rectal Cancer

Before defining early rectal cancer, it is important to define the rectal lesion. According to the American Society of Colon and Rectal Surgeons and the European Society of Medical Oncology (ESMO) guidelines, a rectal lesion is identified as a lesion located within 15 cm of the anal verge in rigid sigmoidoscopy [16,17]. In Japan, the rectum is primarily divided into two sections: above and below the peritoneal reflection, with the upper boundary of the rectum above the peritoneal reflection starting at the lower margin of the second sacral vertebra [5].

In Western countries, early-stage rectal cancer refers to lesions that are confined to the bowel wall, with invasion limited to the submucosa in T1 and the muscularis propria in T2 without lymph node involvement (N0) or distant metastasis (M0) [14]. However, according to the Japanese classification, early carcinomas are classified as Tis and T1 [18]. Approximately 10–15% of all T1 CRC already present with lymph node metastases at the time of diagnosis. Among these, pedunculated T1 CRC cases have a lower risk of lymph node involvement (3–7%) compared to sessile lesions, which carry a risk as high as 28% [19,20].

The presence of adenocarcinoma in a polyp is classified as part of early CRC cancer. The Haggitt classification is a system used to classify pedunculated polyp cancer based on the extent of invasion by cancer cells into the stalk of the polyp and is divided into five levels [21]. Table 1 shows the Haggitt classification.

The Kikuchi classification is another classification system that categorizes non-pedunculated lesions based on submucosal invasion depth, subdividing the submucosa into three distinct layers: up to the upper third of the submucosa (sm1), up to the middle third of the submucosa (sm2), and up to the lower third of the submucosa (sm3) [22,23]. Although it may not be the exact reflection, ESMO corresponds Haggitt’s levels 1–3 to sm1 and level 4 to sm1–3. Assessment of the level of invasion with other prognostic factors is helpful in deciding further management following local excision [16,24].

## 3. Oncological Predictive Factors

Lymphovascular invasion, tumor budding, depth of invasion, high tumor grade, polypoid growth pattern, and location of tumor in the rectum were associated with lymph node involvement [25].

Lymphovascular infiltration refers to the presence of malignant cells within submucosal lymph vessels or blood vessels. Specifically, when tumor cells invade the submucosal lymphatic vessels (L1), this is recognized as a crucial independent predictor for the likelihood of lymph node metastases in early CRC and plays a significant role in assessing prognosis [13,19]. Notably, a positive lymphatic status is associated with a 20% risk of lymph node metastases [26].

The relationship between tumor size and lymph node involvement is debated in the literature. Some studies suggest that rectal cancers in the distal third of the rectum exhibit higher susceptibility to lymph node metastasis [27,28], but others consider it a significant factor in lateral pelvic lymph node metastasis [29,30]. Additionally, research has shown that early-stage rectal cancers located in the lower third of the rectum have a significantly higher risk of lymph node positivity [25,30].

Haggitt’s level 4 tumors are also linked to a high risk of lymph node involvement, whereas in cases of sessile tumor growths, the incidence of lymph node involvement varies from 1–2% for Sm1 lesions, 8% for Sm2 lesions, and 23% for Sm3 lesions [4,30].

To determine further treatment after local resection of early rectal cancer, it is crucial to classify the resected specimen into either low-risk or high-risk categories. A low-risk tumor is defined by grade G1 or G2 tumor budding, no lymphatic invasion, and a size under 3 cm in the rectum. In contrast, high-risk tumors include those with grade G3 or G4, lymphatic invasion, or a size exceeding 3 cm in the rectum [19,31]. This classification is significant, as the likelihood of lymph node metastasis in the low-risk group is 1% or less, compared to up to 23% in the high-risk group [20].

## 4. Diagnostic Investigations

### 4.1. Endoscopy

Endoscopists use optical assessment to determine the likelihood of achieving a curative endoscopic resection of a lesion. Factors such as size, bleeding, granularity, and the non-lifting sign, along with the Paris classification system, should be considered in the evaluation of polyps [32]. Multiple techniques have been introduced to improve the detection of T1 tumors and those with deep submucosal invasion, such as magnifying endoscopy, chromoendoscopy, and narrow-band imaging (NBI). However, despite advancements in endoscopic staging, studies indicate that invasive growth may still go undetected during endoscopic evaluations [33].

### 4.2. Magnetic Resonance Imaging (MRI)

Magnetic resonance imaging (MRI) is the standard imaging modality for locoregional staging of rectal cancer. It facilitates comprehensive pelvic visualization, enabling a precise assessment of the circumferential resection margin and other key prognostic indicators [34,35,36]. MRI has been reported to overstage T1 substage up to 54.7% of patients with T1 rectal cancer [32,37]; however, the current development in high-resolution MRI has been reported to be more efficient in distinguishing T stages. This may offer better management of early rectal cancer by helping select the appropriate excision methods, including organ preservation [38,39].

### 4.3. Endorectal Ultrasound Scan (EUS)

Endorectal ultrasound has been reported to be the preferred method for differentiation between early-stage rectal tumors, specifically T1 and T2 classifications; hence, its diagnostic precision exceeds that of MRI [34,40]. EUS yields favorable outcomes; however, its effectiveness is limited by a decrease in resolution at greater depths and challenges related to stenotic and bulky rectal tumors, as well as its unavailability in some centers, and it is operator-dependent [36].

According to Detering et al., adding EUS to MRI in clinical staging reduces overstaging in early rectal cancer; however, this overstaging percentage can still reach 31% [37].

### 4.4. CT Scan

CT scans of the chest, abdomen, and pelvis are routinely used for CRC staging alongside MRI. CT can detect CRC liver metastases with a sensitivity of 74–84% and a specificity of 95–96% [41]. However, it is less efficient in detecting nodal disease [42] with 76% sensitivity for lymph node staging and 55% specificity in rectal cancer [43].

In cases of inconclusive CT or MRI scan findings suggesting potential metastases, PET scans may be indicated to provide definitive clarification and knowledge that may influence disease management [44]. However, there is not enough evidence to support the use of PET scans routinely in all patients [45].

## 5. Management Options

The gold standard management of rectal cancer is total mesorectal excision (TME), which was introduced by Bill Heald et al. in 1982 to reduce local recurrence. The mesorectum is the adipose and lymphatic tissue surrounding the rectum, which is excised during the TME operation by dissection through the avascular plane between the parietal fascia and the visceral fascia of the mesorectum [13,46].

TME can be performed by open, laparoscopic, or robotic approach. For patients with early-stage rectal cancer, rectal resection with TME may represent overtreatment in certain cases [47]. Radical resection procedures, such as abdominoperineal resection (APR) or anterior resection (AR), are linked to considerable risks, including genitourinary dysfunction, fecal incontinence, permanent stoma, and diminished quality of life [47,48,49].

There is a rising interest in local resection and non-operative treatment of early RC, with some recommendations for a watch-and-wait policy for organ preservation [50]. Several trials, such as GRECCAR 2 [51], TREC [52], and some observational studies, have shown promising results for rectal preservation approaches [53,54,55].

According to Monson et al., transanal local excision is recommended for benign rectal lesions, small neuroendocrine tumors, and low-risk early rectal cancer smaller than 3 cm, involving less than one-third of the rectal wall circumference that has moderately and well-differentiated histology, without evidence of lymphovascular or perineural involvement [17].

If a T1 tumor is incompletely excised in low-risk patients, either local surgical excision or completion ESD should be carried out. In contrast, in high-risk cases, radical oncologic resection should be carried out regardless of whether the initial lesion was completely excised [20].

Several studies have demonstrated that local excision after neoadjuvant chemotherapy or chemoradiotherapy can considerably reduce recurrence rates and is a realistic method to preserve the rectum as an alternative to traditional radical resection [56,57,58].

In endoscopic therapy, patient selection is essential and should be based on tumor size and location. According to Shi et al., there are comparable 3-year survival rates between endoscopic resection and standard resection (SR), which are 93.4% and 93.5%, respectively, and this supports endoscopic resection as an effective alternative in appropriately selected cases with proper surveillance [59].

In early rectal cancer, the local resection treatment options include three types of transanal endoscopic surgery (TES): transanal endoscopic microsurgery (TEM), transanal endoscopic operations (TEO), and transanal minimally invasive surgery (TAMIS), while the endoscopic resection includes endoscopic mucosal resection (EMR), underwater (UEMR), and endoscopic submucosal dissection (ESD) [60].

### 5.1. Endoscopic Resection

For superficial colorectal cancers, the main minimally invasive methods for excision are endoscopic mucosal resection (EMR) and endoscopic submucosal dissection (ESD), with the latter offering a higher rate of en-bloc resection, regardless of tumor size [61].

In Western nations, EMR is still the accepted standard of therapy for rectal lesions smaller than 2 cm in size, as it works well in terms of both efficacy and safety. Nevertheless, EMR necessitates piecemeal resection for lesions larger than 2 cm, which is linked to an elevated local recurrence rate and precludes histological evaluation of the resection margins [62,63]. Additionally, a recent cost-effectiveness analysis indicates that an en-bloc resection strategy using ESD may be more economical than a piecemeal resection approach with EMR for rectal lesions, as it reduces the number of patients needing subsequent radical rectal surgery [64].

Endoscopic submucosal dissection (ESD) was developed originally in Japan in 1995 as a new advanced endoscopic modality for gastrointestinal mucosal lesions that offers a less invasive alternative to surgery for localized disease and was designed to overcome the limitations of EMR [49,62]. A modified needle knife is used for submucosal dissection to facilitate en-bloc resection of colorectal lesions and can be used for rectal lesions more than 2 cm [65]. However, there are some challenges, which include its technical complexity, longer procedure time, and the need for specialized training and equipment. Additionally, ESD involves higher costs and increased risks of bleeding and perforation [49,59].

### 5.2. Transanal Endoscopic Surgery

Transanal endoscopic microsurgery (TEM) was initially developed by Dr. Gerhard Buess in 1983 as a method to remove benign lesions of the mid and upper rectum that were difficult to approach with traditional endoscopic techniques [65,66]. The TEM platform enabled access to the proximal end of the rectum, which was limited in transanal endoscopic procedures. Despite its superiority, implementing TEM is challenging due to high acquisition costs, a long learning curve, and restricted availability in specialist centers [67].

Transanal endoscopic operation (TEO), utilizing high-definition video and panoramic thin-film transistor screens, achieves comparable visual clarity to 3D systems. Surgically, TEO is an advancement of TEM and offers comparable technical and clinical outcomes at a lower cost, according to a Xavier Serra prospective randomized trial [68].

A retrospective cohort study done by Jeonghee Han et al. compared TEO and TEM for local rectal tumor resection. The study included 207 patients who underwent local rectal tumor excision, with propensity score matching yielding 72 patients per group. The study reported that TEO has superior outcomes, particularly for higher rectal tumors, with increased tumor distance (8.0 vs. 4.0 cm), shorter hospital stays, higher negative margin rates, and higher non-fragmented specimen rates while maintaining comparable complication rates [69,70].

TAMIS has been introduced by Atallah et al. as an alternative modality since 2010 due to certain technical issues with TEM. It can be performed under general or spinal anesthesia by using a transanal access platform [71]. TAMIS is a feasible and less expensive alternative to TEM, as demonstrated by short series and case reports since its original description [63].

However, TAMIS has significant drawbacks as the rigid structure of laparoscopic equipment creates a significant hurdle in the limited lumen of the rectum, the requirement for a second surgeon to position the camera outside the anal margin, and the laparoscope’s limited vision. Robot-assisted transanal surgery could be the next step forward for TAMIS, addressing the issues outlined above. Adding a robotic platform to TAMIS can improve oncologic excisions through fine motion scaling, increase dexterity with articulated instruments, make working in small spaces easier, and improve surgeon ergonomics [72].

In a comparison between ESD and TES, Sagae et al. found that endoscopic submucosal dissection (ESD) and transanal endoscopic surgery (TES) are equally effective for early rectal tumors. As a result, clinical management should be guided by factors such as local expertise, equipment availability, and cost considerations. Both surgical and endoscopic transanal resection techniques offer high cure rates with minimal complications, making either approach viable depending on the resources and expertise available [60]. Table 2 compaire the difference between all types of local resection. 

Nodal metastasis and local recurrence rates remain below 5% in low-risk groups after local resection. This rate can increase up to 30% if the tumor size exceeds 3–4 cm or if it is found to be high risk on histology analysis, and in such cases, completion of radical surgery is strongly advocated to significantly reduce the likelihood of disease recurrence [73].

### 5.3. Chemoradiotherapy

The use of neoadjuvant chemoradiotherapy in early rectal cancer management is controversial in the literature. According to NICE guidelines, neoadjuvant chemoradiotherapy is not recommended for patients with T1–T2, N0, and M0 [74].

A randomized controlled trial of 100 patients with cT2N0M0 low rectal cancer compared TES and TME following neoadjuvant chemoradiotherapy (nCRT). Each group included 50 patients and achieved R0 resection. After a median follow-up of 9.6 years, results showed comparable local recurrence and overall survival rates between TES and TME, indicating TES as a viable alternative for selected patients [58].

In contrast, according to Li et al., there was no significant impact of neoadjuvant treatment on overall survival when comparing the overall survival rate of patients with T2 early rectal cancer who underwent TEM with or without neoadjuvant therapy [75].

A systematic review showed that the use of chemoradiation following local excision of T1 lesions carries a median local failure rate, including local recurrence, incomplete resection, and distant metastasis of 10%, and for T2 lesions, the local failure rate can reach up to 25% [73,76].

The wait-and-watch strategy is an option for patients with a clinical complete response after CRT, which demonstrates acceptable oncological outcomes. Apart from local resection, no radical surgery is required, but strict clinical and radiological surveillance is performed until there is evidence of local tumor regrowth. Patients who achieve a complete pathological response have been reported to exhibit oncological outcomes equivalent to radical surgery, with the added benefits of lower rates of permanent stomas and reduced treatment-related morbidity and mortality [11,13].

The role of adjuvant chemoradiation as an alternative to radical resection after local excision in high-risk patients remains uncertain and is currently being investigated in an ongoing clinical trial [73].

### 5.4. Surveillance

The main objective of the surveillance program following colorectal cancer treatment is to enhance survival. Incidence of recurrence following resection includes the liver (33%), lungs (22%), the colon (15%), the rectum (up to 35%), and regional lymph nodes (14%). However, the occurrence of metachronous or new primary cancers is relatively low, at 3% [45].

For all T-stages of rectal cancer, according to the 2011 NICE guidelines, patients who have undergone curative surgical resection for nonmetastatic colorectal cancer should have follow-up to identify local recurrence and distant metastases 4–6 weeks after surgery. The follow-up includes at least two CT scans of the chest, abdomen, and pelvis, as well as serum CEA every 6 months for the first 3 years and a colonoscopy 1 year after the initial surgery. However, the new guidelines from 2020 recommend follow-up with serum carcinoembryonic antigen (CEA) and CT scans of the chest, abdomen, and pelvis for the first 3 years but do not mention the time interval for that [74].

As there are no specific surveillance guidelines for patients with early rectal cancer after local resection, we follow our hospital policy for surveillance. For low-risk groups, including T1 and T2 with no lymph node involvement or distant metastasis and polyps classified as Haggett’s level 1 and 2, follow-up includes a CT scan at 9, 18, and 36 months after treatment, tumor markers every six months for three years, and a colonoscopy at 6 to 12 months after treatment and then annually.

In the high-risk group with lymphovascular involvement, positive margin, or poorly differentiated carcinoma who declined further treatment, follow-up includes CT scans at 9–18 and 36 months, MRI scans every four months for one year, and then every six months for two years. A colonoscopy is performed one year after treatment and then again two years later, while flexibile sigmoidoscopy is conducted at 4, 8, 18, 24, and 30 months.

There are some studies that have reported promising results regarding the use of the circulating tumor DNA (ctDNA) for response prediction after neoadjuvant therapy and surgery, as well as for the detection of recurrence in patients with early-stage and locally advanced rectal cancer [77,78]. Circulating tumor DNA (ctDNA) consists of fragmented single- or double-stranded DNA molecules released by neoplastic cells. Some studies have revealed a significant association between elevated ctDNA concentrations and malignant tumor activity. Although ctDNA detection is feasible in various body fluids, including blood, urine, and saliva, plasma samples are preferred due to their superior suitability for ctDNA analysis [78].

## 6. Discussion

The oncological outcome of local resection of early rectal cancer is debatable in the current literature. Local resection techniques were used primarily for palliative purposes in selected patients with early rectal cancer, mainly elderly patients with significant co-morbidities and high anesthetic risk. A systematic review and meta-analysis done in 2015 revealed that local resection was associated with a decreased five-year overall survival rate and increased risk of local recurrence compared to radical resection [79]. Despite this, the advantage of organ preservation techniques is linked to lower morbidity and improved anorectal function and quality of life [80].

However, a recent systematic review by Fadel et al., which included 12,022 patients, suggested that local resection may achieve similar R0 results in comparison to standard TME, while the recurrence rate and five-year survival can be improved by adopting surveillance methods, such as tumor markers, MRI rectum, endorectal ultrasound, and CTAP [81]. This study included older data, which may have influenced the results due to the lack of current investigative techniques. The inclusion of T3-stage tumors and variability in staging across studies may have also contributed to heterogeneity [81].

A new retrospective cohort study showed that local excision after neoadjuvant CRT appears to be equally effective as radical surgery for individuals with downstaged T1 rectal cancer, despite a higher risk of nodal metastases. On the other hand, adjuvant CT did not enhance the prognosis for individuals with T1 tumors, apart from those under 50 years of age [82].

The American College of Surgeons Oncology Group Z6041 trial was a phase II study involving 84 patients with T2 rectal cancer who received neoadjuvant chemoradiotherapy followed by local excision. Downstaging to T0-T1 was observed in 64% of patients, with 44% achieving a full pathological response. Among 72 patients with an average follow-up of 4.2 years, local recurrence was identified in 4% and distant metastasis in 6%. The 3-year disease-free survival and overall survival were 87% and 96%, respectively [83]. These study results are similar to those of Lezoche et al., which is encouraging for the use of organ-preservation techniques in the near future [58]. The results showed that almost all eligible patients undergoing neoadjuvant CRT had a successful local excision (LE) with negative margins. Despite the dose reduction, the trial found substantial CRT-related toxicity, and post-LE complications were not uncommon, including diarrhea and lymphopenia [84].

The Neoadjuvant Chemotherapy, Excision, and Observation for Early Rectal Cancer (NEO) trial showed that a three-month course of induction chemotherapy with mFOLFOX6 or CAPOX may effectively downstage a large number of individuals with low-risk T1–T2 N0 early rectal cancer. This method allows for well-tolerated, organ-preserving surgical therapies with minimal influence on organ function. The one-year and two-year locoregional relapse-free survival rates were 98% and 90%, respectively, with no reports of distant recurrences or deaths. Furthermore, there was little change in quality of life and rectal function ratings [85].

In the review by Buccafusca et al., he suggested that neoadjuvant therapy for individuals with very small or proximal T2 tumors constitutes overtreatment, resulting in side effects and increased morbidity [45].

## 7. Conclusions

In conclusion, early diagnosis of rectal cancer is crucial for improving patient outcomes, with advances in screening methods and imaging techniques playing a vital role in the detection of early rectal cancer. Management strategies, especially organ-preserving approaches, continue to evolve, offering alternatives to radical surgery for select patients, as these reduce morbidity and improve functional outcomes. However, more randomized controlled trials and studies focused on early rectal cancer (T1–T2) are needed to determine the non-inferiority of the local excision compared to radical surgery.

Furthermore, controversies persist in the literature regarding the optimal treatment pathways, particularly in balancing oncological safety with quality of life. Key areas of debate include the role of neoadjuvant therapy, the accuracy of clinical staging, and the risk of undertreatment. Additionally, limitations such as the variability in diagnostic accuracy, the lack of consensus on surveillance protocols, and the need for long-term data on functional outcomes highlight the challenges in defining standardized guidelines. Future research should focus on refining risk stratification, improving non-invasive diagnostic tools, and optimizing treatment strategies to ensure personalized and effective management of early rectal cancer.

## Figures and Tables

**Table 1:** Haggitt classification.

Level	Depth of Invasion
Level 0	Carcinoma invades the mucosae (carcinoma in situ or intramucosal carcinoma).
Level 1	Carcinoma invades through the muscularis mucosae into the submucosa but is limited to the head of the polyp.
Level 2	Carcinoma invades the level of the neck of the polyp (junction between the head and stalk).
Level 3	Carcinoma invades any part of the stalk.
Level 4	Carcinoma invades the submucosa of the bowel wall below the stalk of the polyp but above the muscularis propria.

**Table 2:** Comparison between EMR, ESD, TEM, and TAMIS.

Features	EMR	ESD	TEM	TAMIS
Cost	Low	Low but requires high expertise	Expensive and limited availability	Low cost
Learning curve	Shorter	Longest due to technical difficulty	Steeper	Shorter than TEM
Platform	Endoscopic	Endoscopic	Rigid platform	Laparoscopic or robot-assisted
Indication	Small benign polyps and superficial cancer T1	Large benign polyps and superficial cancer T1	Large benign polyps and early rectal cancer T1 and T2	Large benign polyps and early rectal cancer T1 and T2
Resection depth	Mucosal	Mucosal and submucosal layer	Full thickness	Full thickness
Resection	Piecemeal	Enbloc resection	Enbloc resection	Enbloc resection
Robotic platform	Not applicable	Not applicable	Not applicable	Applicable
Defect closure	Not indicated	Not indicated	Can be closed or left open	Primary closure
